# *QuickStats:* Rate of Cesarean Delivery, by Maternal Prepregnancy Body Mass Index Category* — United States, 2020

**DOI:** 10.15585/mmwr.mm7048a7

**Published:** 2021-12-03

**Figure:**
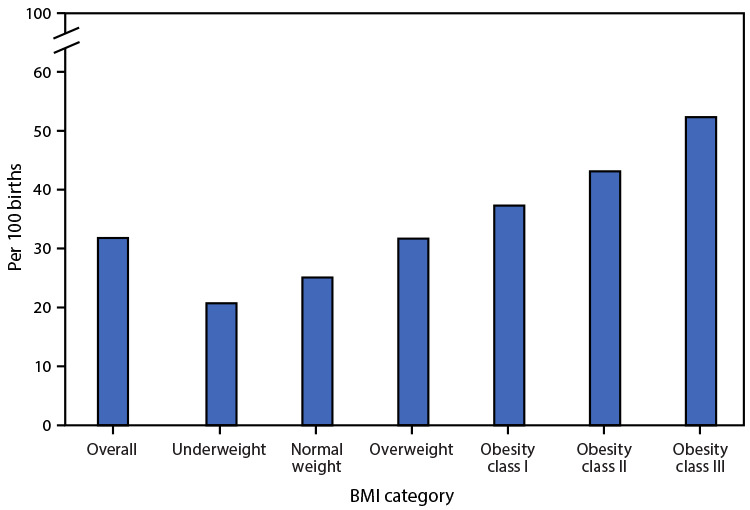
In 2020, 31.8% of live births were to women who had a cesarean delivery. The rate of cesarean delivery was lowest for women who were underweight before pregnancy (20.7%); the rate rose steadily as BMI increased to obesity class III (52.3%). One quarter (25.1%) of women of normal weight had a cesarean delivery.

**Sources:** National Vital Statistics System, natality file. https://wonder.cdc.gov/natality-expanded-current.html; Defining adult overweight and obesity. https://www.cdc.gov/obesity/adult/defining.html#:~:text=Class%203%3A%20BMI%20of%2040%20or%20higher.%20Class,body%20fatness%20or%20the%20health%20of%20an%20individual

